# *Cryptosporidium parvum* screening in young calves with diarrhoea in Sulaymaniyah Governorate, Iraq

**DOI:** 10.17221/60/2024-VETMED

**Published:** 2025-02-26

**Authors:** Saeed Abdulqader, Abdullah Kaya, Hardi Marif, Basim Ali, Dana Ismaeel

**Affiliations:** ^1^Biara Veterinary Centre, Directorate of Veterinary in Sulaimani, Biara, Kurdistan Region, Iraq; ^2^Department of Internal Medicine, Faculty of Veterinary Medicine, University of Yuzuncu Yil, Van, Turkiye; ^3^Department of Clinic and Internal Medicine, College of Veterinary Medicine, University of Sulaimani, Sulaymaniyah, Iraq; ^4^Department of Surgery and Theriogenology, College of Veterinary Medicine, University of Sulaimani, Sulaymaniyah, Iraq

**Keywords:** calves, *Cryptosporidium*, ELISA, PCR, preweaning

## Abstract

The parasitic protozoan *Cryptosporidium parvum* causes cryptosporidiosis in young calves, leading to diarrhoea and financial losses in the farming industry. This study aimed to examine the occurrence of *C. parvum* in preweaning calves suffering from diarrhoea in Sulaymaniyah, Iraq, using both enzyme-linked immunosorbent assay (ELISA) and polymerase chain reaction (PCR) methods. Faecal samples were obtained from 80 young calves categorised into various groups according to age, breed, sex, and geographic origin. Notably, a greater occurrence of *C. parvum* infection was observed in female calves, those in the 5–30 days age group, and those of the Friesian breed. Furthermore, the highest infection rate was reported in the Zarayan region. A strong correlation was observed between the ELISA and PCR findings. The molecular analysis detected both *C. parvum* and *C. ryanae*, with *C. ryanae* documented for the first time in Iraq. *C. parvum* infection considerably affects physiological indicators, particularly in younger calves, including body temperature, heart rate, and respiratory rate. PCR positivity in our study was substantially correlated with dehydration. Overall, this study highlights the need for prompt identification and intervention for the management of *C. parvum* infections in young calves.

Diarrhoea is one of the most common diseases among young calves worldwide (Meganck et al. 2015; Majeed et al. 2022). Calf diarrhoea has a complex aetiology, caused by various infectious agents, such as viruses (rotavirus and coronavirus), bacteria (*Escherichia coli* K99, *Salmonella* spp.), and parasites (*Cryptosporidium parvum*), either alone or in combination with other factors (Icen et al. 2013). This disease leads to significant economic losses due to high morbidity and mortality, poor animal growth, and substantial treatment and veterinary care costs (Meng et al. 2022). *C. parvum* infects a wide range of mammals, including humans (El Ezz et al. 2020), cattle, and small ruminants (Ma et al. 2015). Livestock animals are the main source of *C. parvum*, which causes diarrhoea in weaned calves (Al-Abedi et al. 2022). Cryptosporidiosis affects animal health and production and poses a zoonotic risk for humans through contaminated food and water (Painter et al. 2015). *C. parvum* is an opportunistic pathogen that may be present in diarrhoeic and non-diarrhoeic calves, with higher prevalence in diarrhoeic animals than in non-diarrhoeic. Furthermore, younger animals are more frequently infected with *C. parvum* than older cattle, and a significant association exists between *C. parvum* infection and diarrhoea, as well as the age of the animals (Siddiki and Masuduzzman 2009).

Currently, 22 *Cryptosporidium* species have been isolated from various hosts, including amphibians, fish, reptiles, birds, and mammals. Bovine cryptosporidiosis is mainly caused by *C. parvum, C. bovis, C. ryanae*, and *C. andersoni*, with *C. parvum* and *C. andersoni* significantly more common than the other two species that infect livestock animals (Tarekegn et al. 2021). *C. parvum* is the only zoonotic species often found in weaned calves (aged < 3 months), whereas *C. andersoni* is present in old or mature cattle (Bjorkman et al. 2015).

*C. ovale* is typically detected using various diagnostic methods. Notably, enzyme-linked immunosorbent assay (ELISA) and polymerase chain reaction (PCR) techniques are more sensitive and specific than the microscopic techniques (Al-Robaiee and Al-Farwachi et al. 2014). However, little is known about cryptosporidiosis in Sulaymaniyah.

Therefore, this study aimed to determine the prevalence of *C. parvum* infection in calves with diarrhoea using ELISA and PCR methods. Many investigations have been conducted using various techniques to routinely detect and identify *C. parvum* in animals with diarrhoea. However, studies on the molecular characterisation of this disease in calves with diarrhoea in the surrounding areas of Iraq remain limited.

## MATERIAL AND METHODS

### Sample collection and animal model

Faecal samples were collected from 80 preweaning calves with diarrhoea at five different locations around Sulaymaniyah province. All calves in this study were raised with their mothers and were exclusively fed their mother’s milk. No artificial feeding or supplementation was provided during the trial period. The calves remained with their mothers throughout the observation period, as the housing settings promoted natural rearing practices. The calves were classified into three age groups – first group (5–30 days), second group (31–60 days), and third group (61–90 days) – and five breeds – Friesian, Simmental, Jersey, Holstein, and Local.

Samples were collected directly from calves with diarrhoea in plastic sample containers for ELISA and rectal swabs for PCR. The containers were labelled to identify the entire data collection, and an index card was completed for each animal, indicating the following data: sampling date, address, breed, clinical signs of diarrhoea and dehydration (age, weight, body temperature, heart rate, and respiratory rate), and animal identification number. The samples were then transported to the Sulaymaniyah Veterinary Laboratory in cold containers within hours and stored at –20 °C until analysed.

### DNA extraction

Rectal swab samples were vortexed in 1 ml of phosphate-buffered saline (0.1 M, pH 7), and genomic DNA was extracted from the faecal samples using a DNA extraction kit (Bioprime Co., Daejeon, Republic of Korea) following the manufacturer’s instructions.

### PCR amplification and optimisation

A nested PCR was performed to amplify a fragment of the 18S ribosomal RNA (rRNA) gene using the protocol and primers described previously (Xiao et al. 2001). Briefly, in the first step of nested PCR, an approximately 1.325 bp long PCR product was amplified using *Taq* DNA Polymerase Master Mix (Ampliqon Co., Odense, Denmark) with primers (F1-forward and F1-reverse) in a 0.2 ml PCR tube containing 10 μl 1x master mix, 5 μl DNA sample, 1 μl forward primer (10 pmol), 1 μl reverse primer (10 pmol), and 3 μl ultrapure water to make up the final volume of 20 μl. The conventional PCR machine (Biorad, Hercules, USA) was programmed as follows: initial denaturation at 95 °C for 5 min, followed by 35 cycles of denaturation at 95 °C for 30 min, annealing at 50 °C for 30 s, and extension at 72 °C for 1 min, and final extension at 72 °C for 7 minutes. In the second step of nested PCR, an 826–864 bp product was amplified from 1 μl of the primary PCR mixture using primers (F2-forward) and (F2-reverse), with cycling conditions identical to those of the first step, except for the extension time, which was changed to 40 seconds.

The amplified DNA was then subjected to agarose gel electrophoresis. The gel was prepared by dissolving and boiling the agarose powder or tablet in Tris-borate–ethylenediaminetetraacetic acid 1x buffer using a microwave for approximately 1 min, and after cooling, Simply Safe Dye (5 μl; Eurex Co., Gdaňsk, Poland) was added. The electrophoresis was performed in a specific tank for approximately 45–60 minutes. Finally, the optimisation and identification of amplified DNA from *C. parvum* genes were performed to detect the pathogen in the samples.

### DNA sequencing analysis

Thirty μl of five amplified PCR products of *C. parvum* positive samples were sequenced from both ends using the forward (F2-f) and reverse (F2-r) primers (Macrogen sequencing service, Republic of Korea) for *C. parvum* genotyping.

### ELISA

*C. parvum* antigen in the prepared faecal samples was detected using a commercial ELISA kit (IDEXX Diagnostics, Jericho, USA) following the manufacturer’s instructions. The optical density was measured at 450 nm using an ELISA reader^®^ DAS. The net optical density of each sample was calculated by subtracting the value of each sample from that of the corresponding negative control. The wash concentrate (20×) was diluted to 1 : 20 with distilled water. This solution is hereafter called the “wash solution”, and the conjugate concentrate was diluted 1 : 10 in the N.8 dilution buffer.

### Statistical analysis

Data collection and analyses were performed using Microsoft Excel and SPSS v27. Shapiro–Wilk and Levene tests were used to assess normality and homogeneity, respectively. The Mann–Whitney *U* test was used to evaluate the mean rank of the assessed variables. Additionally, multiple correspondence analysis was used to visualise the correlation between categorical variables.

## RESULTS

### Clinical findings

Eighty samples were collected from five different locations around Sulaymaniyah Governorate in northeastern Iraq. The samples were divided into groups according to age, breed, sex, and geographic location. The highest infection rate was observed in Zarayan (19.4%) among geographical regions and in Friesian calves (22.2%) among breeds. Females were more frequently infected than males. Furthermore, the younger age group (5–30 days) exhibited the highest infection rate (26.8%) (Table 1).

**Table 1:** PCR results for *Cryptosporidium parvum* infection in newborn calves, considering regional distribution, breed, sex, and age

Variables		PCR	
		negative [count (%)]	positive [count (%)]
Location	Halabja	9 (81.8%)	2 (18.2%)
	Khurmal	8 (80.0%)	2 (20.0%)
	Saedsadq	14 (87.5%)	2 (12.5%)
	Sharazwr	10 (83.3%)	2 (16.7%)
	Zarayan	25 (80.6%)	6 (19.4%)
			
Breed	Local	28 (87.5%)	4 (12.5%)
	Friesian	28 (77.8%)	8 (22.2%)
	Holstein	2 (100.0%)	0 (0.0%)
	Jersey	1 (50.0%)	1 (50.0%)
	Simmental	7 (87.5%)	1 (12.5%)
			
Age (days)	(5–30)	30 (73.2%)	11 (26.8%)
	(31–60)	25 (92.6%)	2 (7.4%)
	(61–90)	11 (91.7%)	1 (8.3%)

### ELISA

Of the 80 faecal samples, 12 samples (15%) were positive for *Cryptosporidium* oocysts using ELISA, whereas 14 (17.5%) samples tested positive using nested PCR (Table 2). A kappa value of 0.82 with a *P*-value of 0.000 between PCR and ELISA demonstrated a highly significant and strong agreement between these detection methods, indicating the dependability of the results from both tests.

**Table 2:** Result of samples examined by nested PCR and ELISA

Method	No. of samples	Positive	Negative	Kappa test (*P*-value)
PCR	80	14 (17.5%)	66	12/80 (15%)
ELISA	80	12 (15%)	68	

No significant weight difference was observed in the positive and negative cases among breeds, with all *P*-values > 0.05. However, a statistically significant difference in body temperature was observed between positive and negative cases in the Local and Friesian breeds (*P* = 0.009 and *P* = 0.003, respectively), indicating a possible link between illness and decreased body temperature, particularly in these breeds. A significant difference in heart rate was also observed between positive and negative cases of the Local breed (*P* = 0.002), suggesting a possible association between the disease and reduced heart rate in this breed. In contrast, the *P*-value for the Friesian breed approached significance at 0.059, warranting further investigation. A statistically significant difference in the respiratory rate was observed between positive and negative cases of the Local breed (*P* = 0.006), whereas no other breeds exhibited similar results (Table 3).

**Table 3:** Effect of *Cryptosporidium parvum* infection on the physiological characteristics of young calves of different breeds

Breed	Parameter	Positive	Negative	*P*-value
Local	weight (kg)	25 (31.2 ± 19.1)	30 (31.4 ± 9.4)	0.4
	body temperature (°C)	38.2 (37.7 ± 1.2)	39.1 (39.1 ± 0.9)	0.009
	heart rate (beats/min)	65 (66.8 ± 5.7)	110 (115.8 ± 27.8)	0.002
	respiratory rate (breath/min)	88.5 (87.5 ± 41.6)	33 (37.1 ± 13.3)	0.006
				
Friesian	weight (kg)	32.1 (34.4 ± 12.7)	40 (42.2 ± 12.9)	0.11
	body temperature (°C)	38.1 (38.3 ± 0.5)	39.1 (39.2 ± 0.8)	0.003
	heart rate (beats/min)	88 (90.3 ± 9.9)	107.5 (110.9 ± 27.5)	0.059
	respiratory rate (breath/min)	48 (53.6 ± 9.6)	38.5 (45.8 ± 20.5)	0.083
				
Holstein	weight (kg)	–	47.5 (47.5 ± 10.6)	–
	body temperature (°C)	–	38.6 (38.6 ± 0.9)	–
	heart rate (beats/min)	–	118 (118 ± 8.5)	–
	respiratory rate (breath/min)	–	35 (35 ± 4.2)	–
				
Jersey	weight (kg)	50 (50)	50 (50)	1
	body temperature (°C)	37.5 (37.5)	39.1 (39.1)	0.31
	heart rate (beats/min)	98 (98)	98 (98)	1
	respiratory rate (breath/min)	43 (43)	24 (24)	0.31
				
Simmental	weight (kg)	50 (50)	40 (39.3 ± 15.7)	0.5
	body temperature (°C)	37.8 (37.8)	38.9 (38.9 ± 0.74)	0.27
	heart rate (beats/min)	96 (96)	108 (101.6 ± 15.9)	0.82
	respiratory rate (breath/min)	47 (47)	37 (44.1 ± 19.7)	0.82

Data represent median (mean ± standard deviation); Test: Mann–Whitney *U* test; Positive: Positive PCR results for *C. parvum*, Negative: Negative PCR results for *C. parvum*

The *P*-value indicates a statistically significant relationship between positive and negative results for each breed at the 0.05 level of significance

A statistically significant difference in body temperature was observed between negative cases of the Local breed and both positive and negative cases of the Friesian breed. However, no significant differences in body temperature were observed between the other groups. Similarly, a marked difference in heart rate was detected between the positive and negative cases of the Local breed. Furthermore, significant differences in positive cases were observed among the Friesian breed compared with all other groups. Moreover, negative cases of the Simmental breed differed significantly from positive cases of the Local breed.

No significant variation in weight was observed between the negative and positive cases across age groups, as all *P*-values were > 0.05. However, a significant difference in body temperature (*P* = 0.001) and respiratory and heart rates (*P* = 0.014 0 and *P* = 0.001, respectively) was observed between the negative and positive cases in the 5–30 days age group but not in the other age groups. Additionally, the heart rate in the 31–60 days age group exhibited a trend toward significance (*P* = 0.052), indicating a potential association (Table 4).

**Table 4:** Differences in the effect of *Cryptosporidium parvum* on some physiological parameters between the different age groups of calves

Age group	Parameters	Positive	Negative	*P*-value
5–30 days	weight (kg)	30 (32.2 ± 11.7)	34 (33 ± 9.9)	0.75
	body temperature (°C)	37.9 (37.9 ± 0.77)	39.1 (39.1 ± 0.7)	0.001
	heart rate (beats/min)	90 (87.2 ± 13.9)	116 (118 ± 29.2)	0.001
	respiratory rate (breath/min)	48 (63.2 ± 30.3)	37 (43.3 ± 20.8)	0.014
			
31–60 days	weight (kg)	43 (42.5 ± 24.7)	35 (37.6 ± 11.3)	0.85
	body temperature (°C)	38.6 (38.5 ± 0.21)	39.1 (39 ± 0.9)	0.26
	heart rate (beats/min)	74 (74 ± 16.9)	108 (104 ± 18.5)	0.052
	respiratory rate (breath/min)	56 (55.5 ± 17.7)	36 (39.6 ± 14.9)	0.137
			
61–90 days	weight (kg)	60 (60)	50 (50 ± 15.2)	0.46
	body temperature (°C)	38.5 (38.5)	39.0 (39.1 ± 0.8)	0.19
	heart rate (beats/min)	75 (75)	110 (113 ± 29.5)	0.19
	respiratory rate (breath/min)	62 (62)	34 (39.1 ± 13.8)	0.18

Data represent median (mean ± standard deviation); Test: Mann–Whitney *U* test; Positive: Positive PCR results for *C. parvum*, Negative: Negative PCR results for *C. parvum*

The *P*-value indicates a statistically significant relationship between positive and negative results for each age group at a significance level of 0.05

Overall, this condition appeared to affect body temperature, heart rate, and respiratory rate more significantly than weight. Furthermore, this disease exhibited a more noticeable impact on specific breeds and in various sex and age categories, particularly in the youngest age group. Therefore, it affects the physiological parameters of younger animals more significantly, varying amongbreeds.

**Figure 1:**
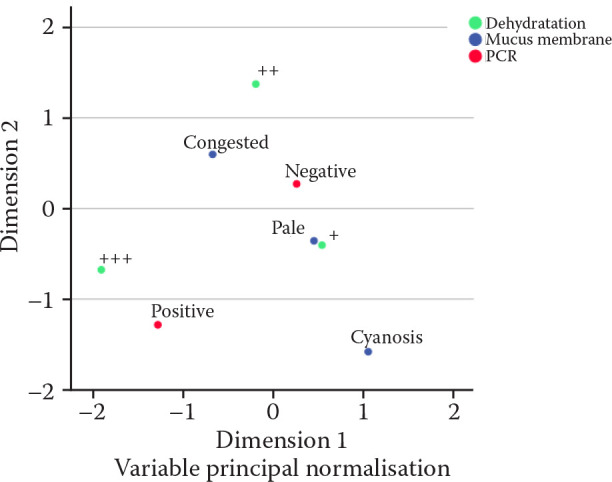
Association between clinical symptoms (dehydration and mucus membrane state) and PCR results

Our findings indicate a correlation between dehydration and a positive PCR result, implying a simultaneous increase in dehydration. However, the presence of mucosal symptoms such as “congestion”, “paleness”, and “cyanosis”, along with the PCR test findings, indicates that they may not be correlated with the PCR result. Specifically, dehydration substantially affected the dataset variance, suggesting it could be a key indicator of *C. parvum* infection in newborn calves. Accordingly, *C. parvum* identification in young calves can be more effectively performed by assessing dehydration rather than checking the colour of the mucus membrane. Therefore, veterinarians should prioritise assessing hydration status early during the clinical examination. These findings can be used to guide veterinary healthcare professionals in identifying key signs to monitor in young calves with diarrhoea, potentially leading to early intervention and improved outcomes (Figure 1).

### Molecular analysis

#### DNA AMPLIFICATION

Of the 80 samples screened using nested PCR, 14 were positive for *Cryptosporidium* oocysts and 66 were negative.

#### DNA SEQUENCING

**Figure 2:**
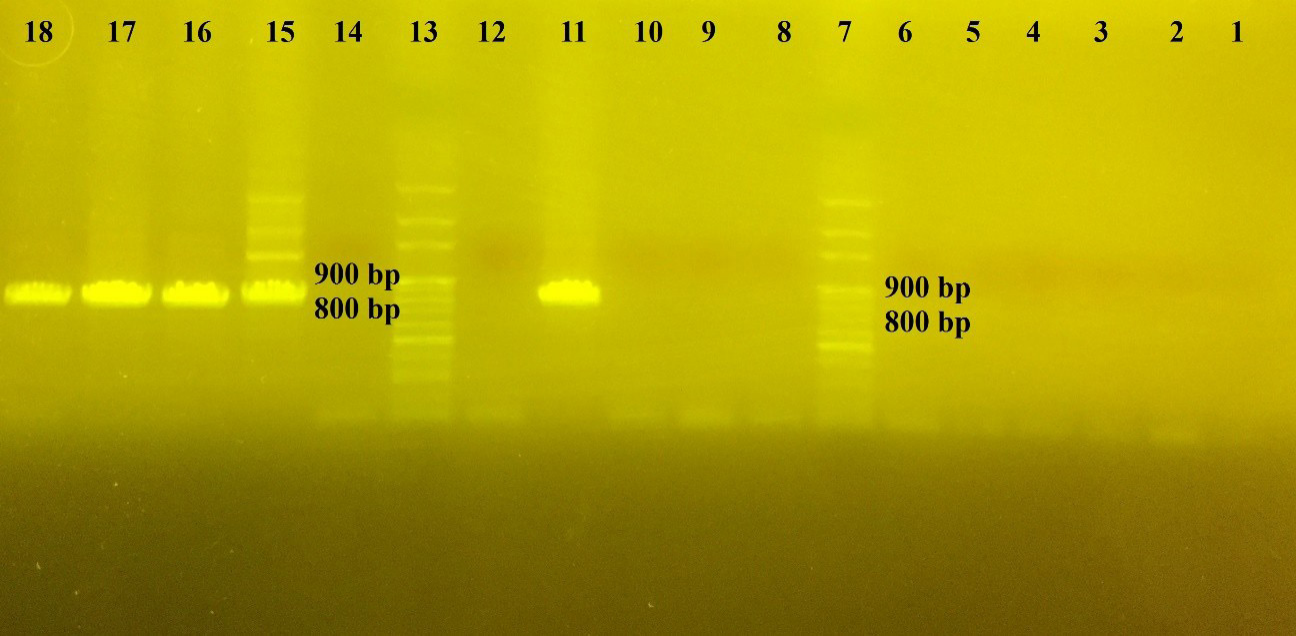
*Cryptosporidium* PCR results based on the 18S rRNA gene after the second overlapping round of PCR 826–886 bp. Samples 1, 2, 3, 4, 5, 6, 8, 9, 10, 12, and 14 are negative; 7 and 13 are DNA size markers; and 11, 15, 16, 17, and 18 are positive

**Figure 3:**
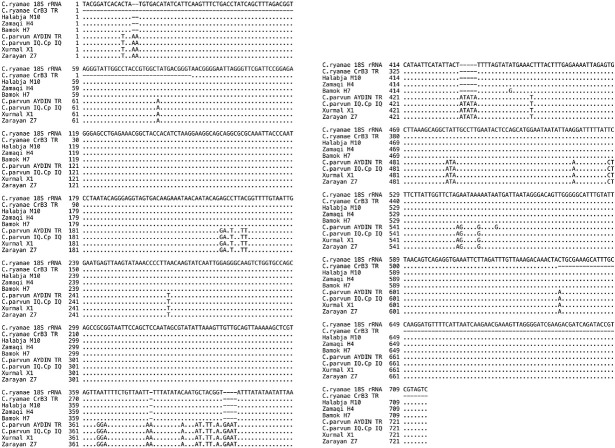
Multiple sequence alignment of five sequences generated in this study with sequences published in GenBank, focusing on sequences from Iraq

**Figure 4:**
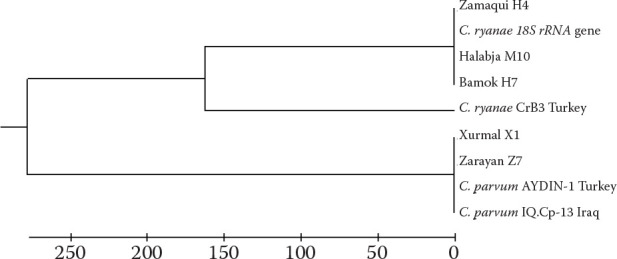
Phylogenetic tree of the analysed sequences showing three sequences (ZamaqiH4, HalabjaM10, and BamokH7) identified as *C. ryanae*, and the other two sequences (XurmalX1 and ZarayanZ7) identified as *C. parvum*

To achieve optimal enrichment of positive PCR primers, five amplified PCR products were sequenced according to the overlapping array (834 bp), and after bioinformatics assembly, final 715 or 727 bp sequences were obtained (Figure 2). Each of the five sequences was compared with published *Cryptosporidium* spp. sequences. BLAST analysis against GenBank sequences revealed that two samples (XurmalX1 and ZarayanZ7) were *C. parvum*, whereas the other three (ZamaqiH4, HalabjaM10, and BamokH7) were *C. ryanae*. CLUSTAL v2.1 and MEGA v6 software programs were used for multiple sequence alignment and phylogenetic tree construction (Figure 3). A phylogenetic tree was constructed using the MEGA v6 software to visualise the grouping characteristics of sequences according to their differences. Notably, all three *C. ryanae* samples clustered together, as did the *C. parvum* samples from Türkiye and Iraq (Figure 4).

## DISCUSSION

The explanation for these results is that 78.5% of all positive samples were from calves under 1 month of age (approximately 1–3 weeks). Furthermore, most calves with diarrhoea (79.38%) were aged < 1 month. Similar results have been reported in previous studies (Rieux et al. 2014; Mammeri et al. 2019), with the highest infection rate of *C. parvum* in calves aged 1–3 weeks. In our study, younger calves (5–30 days age group) were more susceptible to *C. parvum* infection than older calves (31–30 and 60–90 days age groups). A previous study also reported higher prevalence in preweaning calves aged < 2 months (45.8%) than in postweaning calves aged > 2 months (18.5%) (Santin et al. 2004; Diaz et al. 2021), likely because of the immature immune system in young calves. Consequently, higher infection rates are observed in new born, and infection is less common in young calves. One study reported the highest cryptosporidiosis rate of 23.2% in calves aged 1–2 months (23.2%), followed by 19.3% and 16.7% in those aged 0–1 and 2–3 months, respectively (Simsek et al. 2012).

The prevalence of cryptosporidiosis in some studies is less than (17.5%) in other studies (Santin et al. 2004), potentially due to different geographical and environmental factors or different management systems applied by farmers. However, no information about animals and management systems was collected to explain this difference (Muhid et al. 2011). All samples in our study were obtained from live animals, with none collected from private farms. The spread of cryptosporidiosis may be linked to management practices, particularly in cases of overcrowding. Poor hygiene and inadequate management measures are risk factors for diarrhoeal diseases in farms (Diaz-Lee et al. 2011; Felefel et al. 2023).

Animal breed plays a crucial role in infection susceptibility, with local breeds of calves being more prone to infection than other breeds, owing to their physiological structure, requiring a specific environment, management practices, and sanitary conditions. The environment of this geographical region may not be suitable for the Friesian breed but may be preferred by other breeds, such as Jersey and Simmental (Ma et al. 2015).

In this study, sex influenced the morbidity in infected calves, and differences in susceptibility to *C. parvum* were observed between males and females, consistent with a previous study (Santos et al. 2023). *C. parvum* mainly infects pre-weaned dairy calves, whereas *C. bovis* and *C. ryanae* are found in older or weaned calves. *C. bovis* was detected in calves at 5 weeks of age and *C. ryanae* at 15 weeks of age (Aberg et al. 2020), whereas other studies demonstrated earlier excretion of these species (Rahi et al. 2013).

Although different methods (microscopic, serological, and molecular) are used for screening *C. parvum* (Cho et al. 2013; Garro et al. 2021), nested PCR and ELISA are used more widely. However, distinguishing *C. bovis* and *C. ryanae* morphologically from *C. parvum* is difficult, as *C. ryanae* is similar to *C. parvum* and *C. bovis* but is smaller. Consequently, molecular analysis involving DNA sequencing of the nested PCR products is necessary to differentiate these three intestinal *Cryptosporidium* spp. (Gattan et al. 2023).

In this study, only *C. parvum* and *C. ryanae* were detected in calves early in life. Other studies have reported *C. parvum*, *C. bovis*, and *C. ryanae* in young calves at 3 weeks of age. Furthermore, both *C. bovis* and *C. ryanae* have been reported in all cattle age groups in several locations (Rieux et al. 2014).

The clinical signs of the three calves infected with *C. ryanae* were different from the other two infected by *C. parvum*. For example, calves infected with *C. ryanae* exhibited no dehydration and had slight diarrhoea, whereas the other two calves had severe dehydration and diarrhoea. A previous study reported *C. ryanae* infection in calves exhibiting subclinical signs, whereas *C. parvum* was the predominant species in diarrhoeic calves (Rieux et al. 2014). Neonatal calves are more susceptible to *C. parvum* and *C. bovis* infection than *C. ryanae* infection (Silverlas et al. 2013). The colour of the faeces of three calves infected with *C. ryanae* was green, whereas that of the other two calves was yellow. Therefore, we could separate these into genotypes among all positive samples based on the recorded clinical signs. To date, *C. ryanae* has not been reported in Iraq. This is the first study to report *C. ryanae* in neonatal diarrhoeic calves in Iraq. The lack of reports on *C. ryanae* in previous epidemiological studies in Iraq is likely due to the use of older genotyping tools. The reliable separation between *C. parvum* and *C. ryanae* is often problematic due to very slight changes in their 18S rRNA-based RFLP patterns (Khan et al. 2010; Das et al. 2019).

This study provides important insights into the epidemiology and molecular characteristics of *Cryptosporidium* in preweaning calves in Sulaymaniyah, Iraq. Our findings emphasise the importance of *C. parvum* as a primary cause of diarrhoea in young calves, warranting reliable diagnostic techniques, such as ELISA and PCR, to ensure a precise diagnosis. The discovery of *C. ryanae* in this area enhances our understanding of the variety and distribution of *Cryptosporidium* species. Furthermore, the correlation between dehydration and positive PCR results implies that evaluating the hydration status should be prioritised in the clinical examinations of calves with diarrhoea. Overall, our findings may aid in the development of focused approaches to prevent and manage *Cryptosporidium* infections in young calves, reducing economic losses and enhancing animal health.
